# A hybrid data-driven solution to facilitate safe mud window prediction

**DOI:** 10.1038/s41598-022-20195-7

**Published:** 2022-09-21

**Authors:** Ahmed Gowida, Ahmed Farid Ibrahim, Salaheldin Elkatatny

**Affiliations:** Department of Petroleum Engineering, King Fahd University of Petroleum & Minerals, Dhahran, 31261 Saudi Arabia

**Keywords:** Solid Earth sciences, Engineering

## Abstract

Safe mud window (SMW) defines the allowable limits of the mud weights that can be used while drilling O&G wells. Controlling the mud weight within the SMW limits would help avoid many serious problems such as wellbore instability issues, loss of circulation, etc. SMW can be defined by the minimum mud weight below which shear failure (breakout) may occur (MW_BO_) and the maximum mud weight above which tensile failure (breakdown) may occur (MW_BD_). These limits can be determined from the geomechanical analysis of downhole formations. However, such analysis is not always accessible for most drilled wells. Therefore, in this study, a new approach is introduced to develop a new data-driven model to estimate the safe mud weight range in no time and without additional cost. New models were developed using an artificial neural network (ANN) to estimate both MW_BO_ and MW_BD_ directly from the logging data that are usually available for most wells. The ANN-based models were trained using actual data from a Middle Eastern field before being tested by an unseen dataset. The models achieved high accuracy exceeding 92% upon comparing the predicted and observed output values. Additionally, new equations were established based on the optimized ANN models’ weights and biases whereby both MW_BO_ and MW_BD_ can be calculated without the need for any complicated codes. Finally, another dataset from the same field was then used to validate the new equations and the results demonstrated the high robustness of the new equations to estimate MW_BO_ and MW_BD_ with a low mean absolute percentage error of 0.60% at maximum. So, unlike the costly conventional approaches, the newly developed equations would facilitate determining the SMW limits in a timely and economically effective way, with high accuracy whenever the logging data are available.

## Introduction

Geomechanics is among the sciences that represent a cornerstone when it comes to planning and optimization of drilling and development of petroleum fields. It comprises studying the mechanical properties of the rocks in addition to the distribution of the in-situ stresses in the vicinity of the wellbore. Careful estimation of such information is considered a key factor that could help avoid a broad range of costly issues that may be encountered while drilling, completion, and stimulation operations. Geomechanics of the subterranean formations can be studied by building geomechanical models that mimic the in-situ geomechanical state of the formations^1^. This could contribute to the technical investigation of different processes and in turn facilitate cost reduction. The geomechanical model can be constructed using conventional logging data, i.e., formation density logs, porosity logs, acoustic logs, etc. in addition to some field tests and core-based lab experiments for calibration purposes. Wellbore stability is considered among the main concerns during the drilling process. Two common issues relate to wellbore stability: tensile failure (fractures) or wellbore breakdown (collapse). As a result, many problems may occur loss of circulation, pipe sticking, washouts, etc. Therefore, care should be taken while well planning for determining the safe mud window to avoid such costly and wasting-time events. The safe mud window is typically determined in light of the in-situ stress state of the downhole formations^2^. The formation stresses are described in terms of both magnitude and direction whereby the probability of wellbore-instability problems can be evaluated. Accordingly, accurate data on the in-situ stress configuration are usually required along the well depth to come up with viable solutions for potential instability issues while drilling. This can be attained by deploying the optimal mud weight that exists within the range of the predefined safe mud window (SMW)^3^. SMW can be determined by applying geomechanical modeling based on well data including the in-situ stresses, pore pressure, and rock mechanical properties coupled with suitable rock failure criteria^4^. However, such analysis is considered costly and time-consuming which limits its accessibility for most of the drilled wells.

Recently, similar to different industries (Haghighat and Li, 2021), there is a growing need for automated intelligent systems that can make use of the availability of enormous data during different operations in the O&G industry. These systems are based on different machine learning approaches that aim at modeling and estimating different key parameters in a cost-effective and time-efficient way^5^. Many studies in the literature have employed machine learning approaches for estimating different geomechanics-related parameters that would be used for designing SMW. In 2012, Rabbani et al. developed a model using neural networks to predict the unconfined compressive strength (UCS) which is one of the key parameters in earth geomechanical modeling, using the well log data such as density, porosity, etc. from an oilfield in southern Iran. Pereira et al. (2013) introduced a model based on decision trees to predict the appropriate mud weight for safely drilling salty formations. They used drilling records (e.g., date/depth, observed mud weight), lithology types encountered and the records of the incidents, e.g., pipe sticking due to formation swelling. Tabaeh and Mohammad (2016) also developed a neural network-based model to predict the shear wave velocity based on the logging data. Then they incorporated this information into a new workflow to estimate the least principal stresses in a tectonically active region. Zhou et al. (2016) developed a new model to predict the adequate equivalent circulating density (ECD) to control the downhole pressures under HPHT conditions. They used the type of the drilling mud, fractions of oil and water phases in the used mud in addition to the formation conditions of pressure and temperature as input features to their developed neural network. Okpo et al. (2016) investigated wellbore instability in a Nigerian field by deploying a neural network approach. They used the mud properties, formation stresses, bottom-hole pressure, pore pressure, and borehole diameter as inputs to determine the stability of the downhole formations. Zahiri et al. (2019) studied the safe mud window by developing a mechanical earth model of an Iranian field. They then employed the neural network approach to predict the failure criterion based on the logging data in a black-box model. Recent studies on SMW have applied rock wall stability/failure assessment models under tensile, shear and in situ stresses combined with ML models^6,7^. Though Phan et al.^7^ reported high prediction accuracy, they used several input parameters that are not always accessible for most oilwells such as principal stresses, elastic modulus, Poisson’s ratio, cohesion strength, tensile strength. The need for such parameters limits the applicability of these models in the wells where such data are not available. In^6^, Abbas et al., introduced the application of ML to predict SMW from logging data. The study lacks any error metrics that are essential to quantify the prediction error and the model’s accuracy. Based on the review of the literature, the existing intelligent models applied to predict SMW leave scope for improvements in accuracy and applicability which should beneficially contribute to safe and optimized drilling operations.

Therefore, in this study, the safe mud window (SMW) has been investigated using a machine learning approach (artificial neural network, ANN). SMW has been determined by studying the minimum and maximum mud weight limits beyond which either shear or tensile failure may be encountered respectively. This study aims at predicting the SMW from the logging data directly using an ANN-based model for a Middle Eastern field. Besides, the developed ANN model has been presented in a white-box mode by establishing new equations that imitate the processing of the developed ANN.

The new approach was established based on the analysis of the geomechanical data to enable the direct determination of the safe range of drilling mud weights whenever the logging data are available. Unlike the conventional high-priced approach, the newly developed equations would facilitate estimating the MW_BO_ and MW_BD_ directly from the logging data in an economic and time-effective way. This, in turn, would help the petroleum engineers design adequate mud properties whereas many wellbore instability problems can be avoided. In addition, drilling planning and optimizations could be improved further.

In the upcoming section, the methodology adopted for this study is illustrated followed by a detailed demonstration of the models’ development process and their optimization. Finally, the results of this research are discussed in the discussion section with summarized outcomes in the conclusions section.

## Materials and methods

### Methodology

Rock mechanics refers to the science of investigating the geomechanical behavior of the rocks either under elastic or failure states. These behaviors are expressed in terms of both elastic parameters, i.e., elastic moduli (E), Poisson’s ratio (PR), etc. in addition to failure parameters, i.e., unconfined compressive strength (UCS), friction angle (Ø), tensile strength (T_s_), etc. Such parameters are basically incorporated while developing the mechanical earth model (MEM) whereby the geomechanical behavior of the subterranean formations can be studied. Firstly, data have been collected for constructing MEM from three wells within a Middle Eastern field. Three groups of data were included, these are:

- Petrophysical logging data, e.g., formation bulk density (RHOB), sonic data (DTC and DTS), gamma-ray log (GR), neutron logging (NPHI), and caliper logs (CALI).
- Core data are based on the experimental tests conducted on the retrieved core samples. These data are usually used for model validation and calibration. The calibration means identifying the possible relations among the dynamic elastic properties that are estimated from the well logs and the corresponding static ones that are measured in the lab.
- In-drilling data that are collected during the drilling operation such as mud losses, and wellbore instability issues reporting.

Secondly, the dynamic elastic parameters (E and PR) have been estimated using the petrophysical data and then calibrated into the corresponding static ones using the available core data. This led to having continuous profiles of static E and PR along with the depth of the studied wells. Next, the failure parameters (UCS, T_s,_ and Ø) have been correlated to obtained well logs to get them in a continuous-profile form. These steps are essential for developing MEM that effectively represents the geomechanical behavior of the downhole formations.

Then, the stress field (e.g., overburden stress S_v_, maximum and minimum horizontal stresses Sh_min_ and Sh_max_) has been determined using the dual poroelastic models. More information on the determination of the formation stresses for the field under study are available in the published studies^8–10^.

When a well is drilled into a formation, stressed solid material is removed. The borehole wall is then supported only by the hydrostatic pressure of the drilling mud in the hole^11^. As this fluid pressure generally does not match the in-situ formation stresses, there will be a stress redistribution around the well, known as the post-drilling stress field or induced stress field. The secondary induced stresses: hoop stress (S_θθ_), radial stress (S_rr_), and axial stress (S_zz_) have been calculated based on the Sh_min_ and Sh_max_ using the following equations^2^:

1 $${{S}_{\theta \theta }}_{max}={{3 S}_{H}}_{max}-{{S}_{h}}_{min}-{P}_{w}-{P}_{f}$$

2 $${{S}_{\theta \theta }}_{min}=3{{S}_{H}}_{min}-{{S}_{h}}_{max}-{P}_{w}-{P}_{f}$$

3 $${{S}_{zz}}_{max}={S}_{v}+2 PR\left({{S}_{h}}_{max}-{{S}_{h}}_{min}\right)-{P}_{f}$$

4 $${{S}_{zz}}_{max}={S}_{v}-2 PR\left({{S}_{h}}_{max}-{{S}_{h}}_{min}\right)-{P}_{f}$$

5 $${S}_{rr}={P}_{w}-{P}_{f},$$

*${P}_{w}$: Hydrostatic mud pressure, *${P}_{p}$: Formation pore pressure.

There are two main scenarios through which the borehole may fail, depending on the relative magnitudes of these secondary stresses at the borehole wall. First, a shear failure that results from using underweighted drilling mud compared to the formation pressure, leads to breakout failure. Second, a tensile failure that results from using overweighted drilling mud, leads to induced fractures (breakdown failure) and hence partial or complete losses.

At this stage, wellbore stability analysis has been undertaken to determine SWM by estimating three key parameters:

- Pore pressure (P_f_) that are usually collected during well testing.
- Breakout mud weight (MW_BO_)
- Breakout mud weight (MW_BD_).

For determining the breakout mud weight (MW_BO_), in practice, different permutations of the stresses S_θθ_, S_rr,_ and S_zz_ should be considered while studying the mechanical stability of the region and then the largest borehole mud pressure at failure would be selected as a breakout failure criterion to determine MW_BO_. The equations for these permutations are summarized in (Table 1)^11^.

**Table 1:** Borehole shear failure (Breakout) criterion to determine MW_BO_ for borehole stability.

Case	Stress state	Borehole failure occurs when
1	${S}_{\theta \theta }\ge {S}_{zz}\ge {S}_{rr}$	${P}_{w}\le \frac{A-C}{1+\mathrm{q}}$
2	${S}_{zz}\ge {S}_{\theta \theta }\ge {S}_{rr}$	${P}_{w}\le \frac{B-C}{1+\mathrm{q}}$
3	${S}_{zz}\ge {S}_{rr}\ge {S}_{\theta \theta }$	${P}_{w}\le A-C-qB$

Where; $A={{3 S}_{H}}_{max}-{{S}_{h}}_{min}$, $B={S}_{v}+2 PR\left({{S}_{h}}_{max}-{{S}_{h}}_{min}\right)$, $C=UCS-{P}_{p}\left({\mathit{tan}}^{2}\left(\beta \right)-1\right)$, $q={\mathit{tan}}^{2}\left(\beta \right)$, where, A, B, and C are constants that are calculated based on other parameters, i.e., Sh_min_, Sh_max_, S_v_ and PR. UCS refers to the uniaxial compressive strength and β denotes the failure angle.

In addition, the tensile failure may be encountered when a significantly high mud weight is used that may exceed the value given by Eq. (6). This MW_BD_ value has been considered as the breakdown failure criterion^1,11^. Finally, the MW_BD_ and MW_BD_ profiles were generated and used as the outputs for the proposed models.

6 $${MW}_{BD}={{3 S}_{H}}_{max}-{{S}_{h}}_{min}-{P}_{f}+{T}_{s}$$

### Data analysis

#### Data description

In this study, a dataset of 1858 actual observations are gathered from three wells in a Middle Eastern field representing a complex carbonate reservoir. The data included the petrophysical logging data: GR, RHOB, DTC, DTS, and NPHI and the corresponding estimated MW_BO_ and MW_BD_ at the same depth_._ The safe mud weight limits were determined based on the analysis of the developed MEM for the area under study as described in the “Methodology” section to eventually determine the MW_BO_ and MW_BD_ profiles. The data collected from two wells were used for training and testing the model (divided at a certain ratio) while the rest of the data (the third well) have been used for validating the developed models.

#### Data pre-processing and filtration

**Figure 1:**
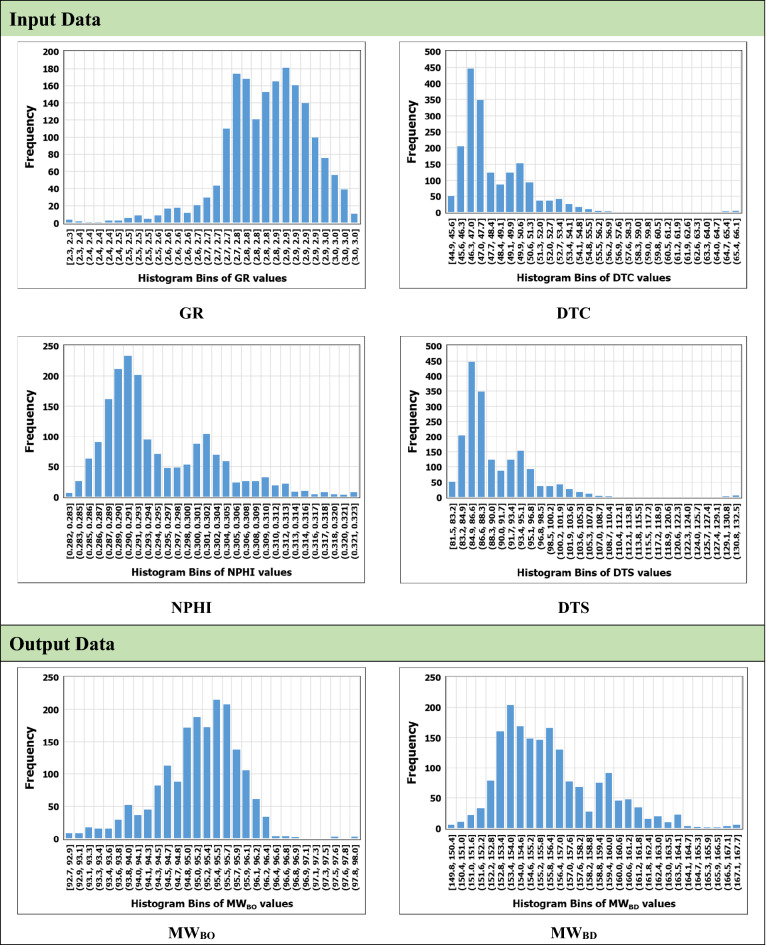
Histogram of the data used in this study.

The prediction accuracy of the AI-based models is significantly affected by the quality of the data used while developing the model. Therefore, the data were pre-processed and filtered using statistical analysis and engineering sense based on the literature. A specially designed Python code was used to remove unreasonable values like zeros and negative values in addition to any missing points. Then, outliers were removed using a box and whisker plot, in which the top whisker represents the upper limit of the data and the bottom whisker represents the lower limit of the data^12^. Any value beyond these limits was considered an outlier and then was removed. These limits were determined using the statistical parameters listed in Table 2 which lists the descriptive statistical summary of the dataset used in this study to reflect its distribution and covered ranges. Figure 1 shows the histogram distributions of the input and output data.

**Table 2:** Descriptive statistical analysis of the dataset used in this study.

Parameter	GR (API unit)	DTC (µs/ft)	DTS (µs/ft)	RHOB (g/cm^3^)	NPHI	MW_BO_ (Ib/ft^3^)	MW_BD_ (Ib/ft^3^)
Minimum	3.38	44.89	81.28	2.32	0.28	92.73	149.82
Maximum	85.79	66.12	132.28	3.04	0.32	98.01	167.71
Mean	29.25	48.55	90.26	2.83	0.30	95.11	156.23
STD	15.12	2.91	7.00	0.11	0.01	0.79	3.11
Skewness	4.12	44.82	81.28	2.38	0.28	92.73	150.15

#### Dimensionality reduction analysis

**Figure 2:**
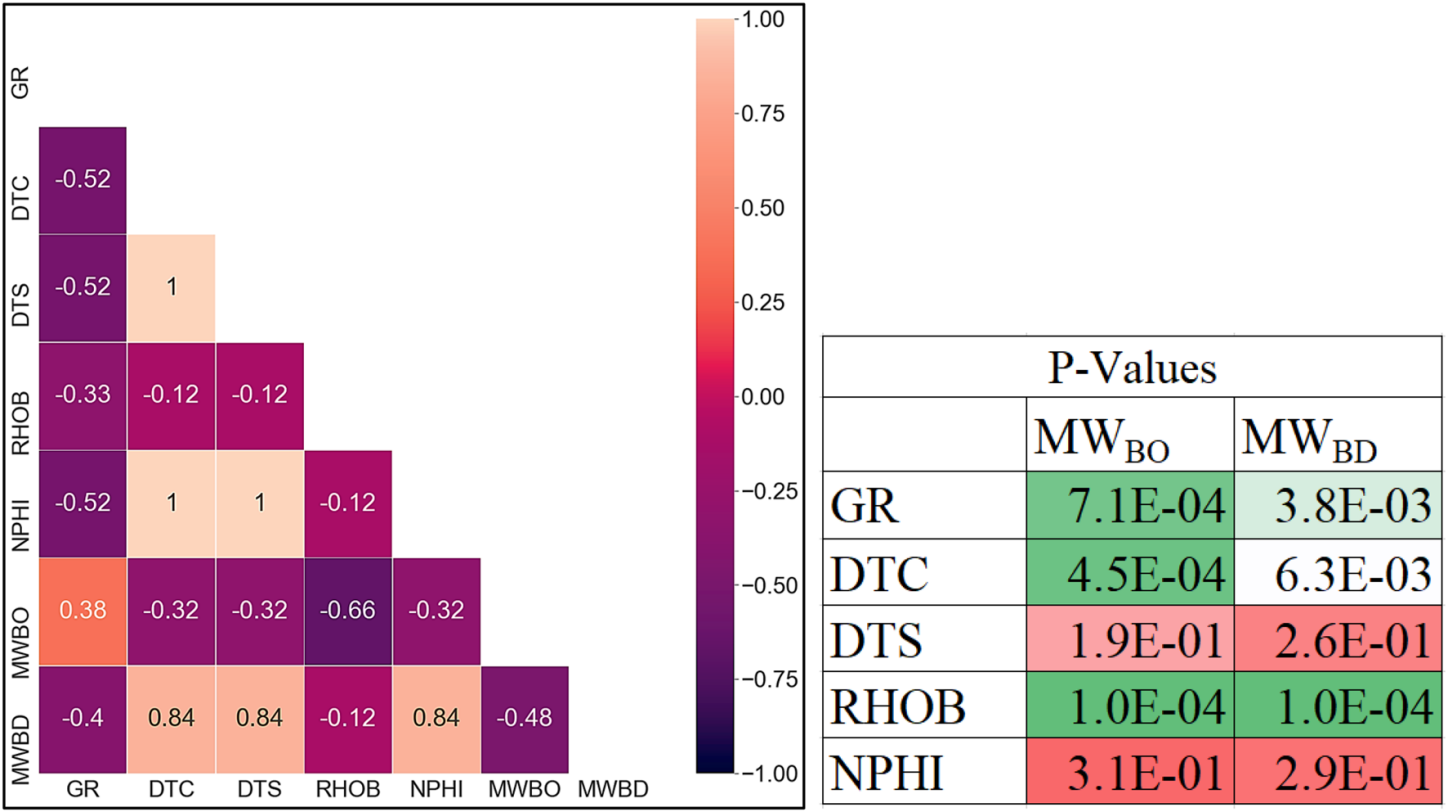
Heat map reflecting the collinearity among the input/output parameter with the P-values associated with the correlation coefficients.

The collinearity between each parameter and others is presented in the heat map shown in Fig. 2, in the way that the darker the color is, the higher degree of collinearity between the two parameters. The values presented in the heatmap represent the Pearson correlation coefficient (R-value) between every two parameters. The correlation coefficient is used to identify how strongly two parameters are linearly related to each other. Its value ranges from − 1 to + 1. A strong direct linear relation is indicated with an R-value of + 1. On the contrary, the R-value of − 1 shows a strong inverse linear relationship between these two variables. While an R-value of zero indicates no linear relationship exists between the two studies' variables. Moreover, Fig. 2 displays P-values associated with the correlation coefficient between the input and the output parameters to examine the significance of the correlation between the input and the outputs in the regression process. The DTC has shown a high correlation coefficient of 1 and 0.95 with DTS and NPHI respectively. Thus, DTC has been only considered as an input feature for the developed model to avoid the redundancy of the input information to the proposed models. These results were also confirmed by the p-values, where p-values were less than 0.05 (that means it is significant) except for the DTS and NPHI. Accordingly, the final set of the selected input features was GR, DTC, and RHOB.

#### Sensitivity analysis

**Figure 3:**
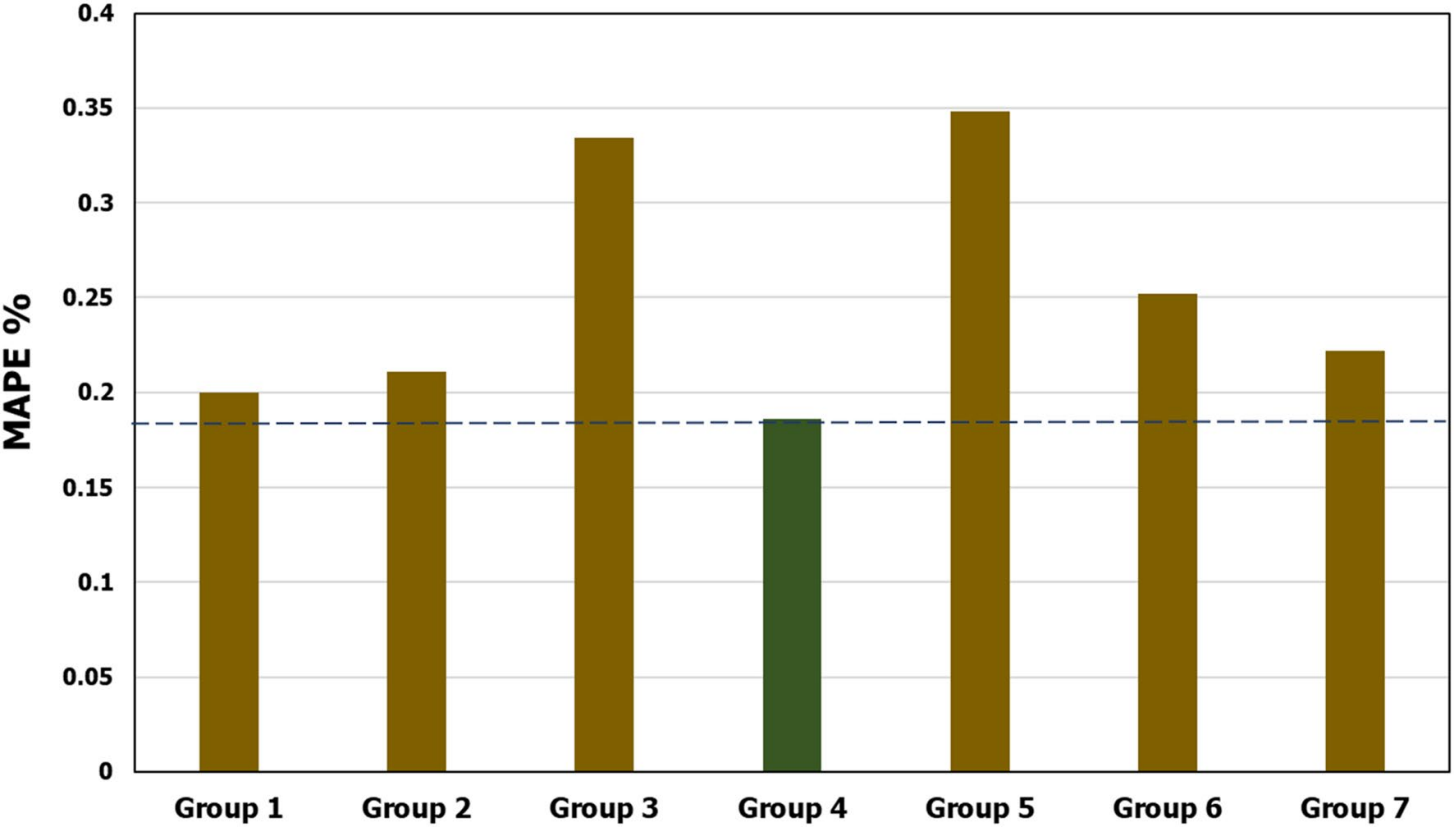
Comparison of the prediction error for the tested input groups.

In addition to studying the collinearity between the parameters to select the most effective ones, a sensitivity analysis has also been conducted. Different trials have been conducted using different sets of input parameters to predict MW_BO_. This step was to get initial insights into the effect of each input parameter on the prediction results. Different groups of input parameters have been tested. These groups are Group 1 (GR, DTC, DTS, RHOB, NPHI), Group 2 (GR, DTC, DTS, RHOB), Group 3 (GR, DTC, DTS), Group 4 (GR, DTC, RHOB), Group 5 (GR, DTC), Group 6 (GR, RHOB) and Group 7 (DTC, RHOB). The results have been evaluated in terms of mean absolute percentage error (MAPE) between the actual and estimated output values. Figure 3 shows a comparison of the prediction error for the tested input groups. The results showed that the combination of GR, DTC, and RHOB (Group 4) yielded the lowest MAPE and hence the best prediction accuracy. This observation agrees with the conclusion of the dimensionality reduction analysis that ended with using GR, DTC, and RHOB combination as inputs for developing the proposed models. Accordingly, this set of inputs has been decided for developing the prosed models.

### Model development

In this research, artificial neural network (ANN) has been applied to develop a new model that can directly predict both MW_BO_ and MW_BD_ based on well-logging data. ANN has been selected due to its recent successful application in petroleum-related geomechanics. Many studies in the literature have reported the application of ANN to predict several geomechanical parameters to be used in the petroleum field context such as UCS prediction^13–15^, elastic moduli, and Poisson’s ratio^16–18^, stress field prediction^8,9,19^, etc. Typically, ANN consists of three basic types of layers: the input layer including GR, DTC, and RHOB, the hidden layer(s), and the output layer comprising MW_BO_ and MW_BD_. Training the network starts with processing the data from the hidden layer through weighted connections to the neurons in the hidden layer to ultimately estimate the outputs^20^. For optimizing the network, *GridSearchCV* function was designed using Python code to iteratively test different parameters for optimizing each algorithm within the predefined ranges by the user, to report the best value of each parameter for each algorithm. The obtained data has been divided into two sets: training and testing. The training set was used to train the model and optimize its hyper-parameters. During the optimization process, the results of the models were internally tested to evaluate the selected hyper-parameters. For each trial, the predictions were evaluated using the R-value and the error between the actual and predicted output values for the training, and testing processes. The objective of this step is to identify the hyper-parameters that could achieve the lowest possible prediction error through many iterative trials. Afterwards, the model with the optimized hyper-parameters was evaluated using the testing set to estimate the generalization error of the optimized model^21^.

**Figure 4:**
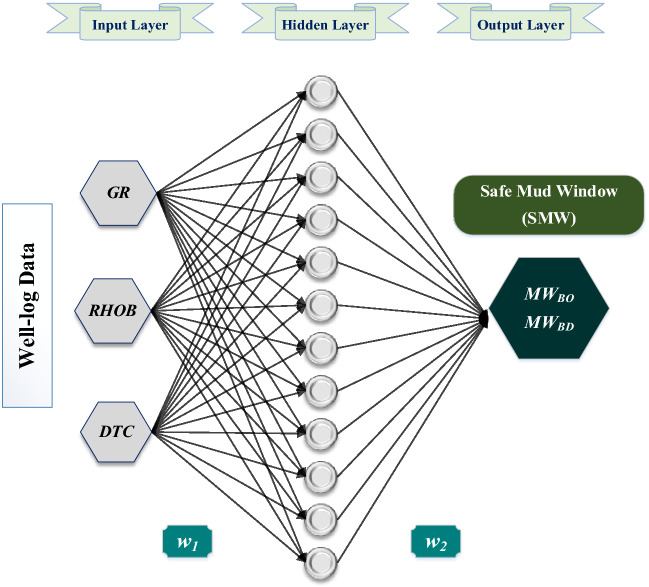
Typical schematic of the developed ANN architectures.

**Figure 5:**
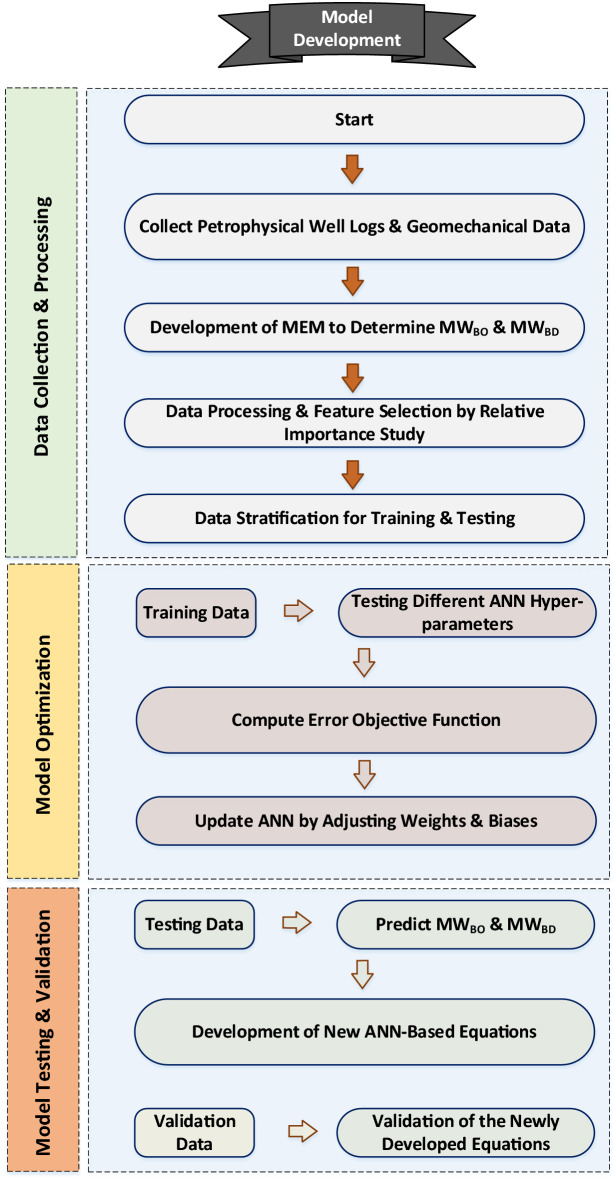
Flowchart for developing the proposed new ANN-Based equations.

The prediction results have been evaluated in terms of correlation coefficient (R), MAPE, mean squared error (MSE), and root means squared error (RMSE) between the observed and predicted outputs. The mathematical formulas for the evaluation metrics are stated in Appendix A. Table 3 lists the predefined options of the ANN parameters (e.g., training algorithm, transfer function, number of neurons, number of hidden layers, and learning rate) that have been tested during the tuning process. Figure 4 shows a typical schematic architecture of the developed ANN model. Figure 5 shows the workflow followed in this study to develop the proposed equations.

**Table 3:** The tested options for optimizing the developed ANN models.

Parameter	Tested options/ranges			Optimized parameters	
				MW_BO_ model	MW_BD_ model
Number of hidden layers	1–4			1	
Number of neurons in each layer	5–40			10	7
Split Ratio	70–90% (For training set) The rest was used for testing			70 (train) : 30 (test)	
Training algorithms	trainlm	trainbr	trainrp	trainbr	
	trainscg	traincgb	traincgf		
	traincgp	trainoss	traingdx		
Transfer function	tansig	logsig	elliotsig	tansig	
	radbas	hardlim	satlin		
Learning rate	0.01–0.9			0.12

## Results and discussion

### Safe mud window prediction

Two models have been developed using ANN to predict both MW_BO_ and MW_BD_. The developed models have been optimized by selecting the tuning parameters that yielded the lowest MAPE and the highest R between the observed and predicted output sets.

For MW_BO_, the optimized model comprised one hidden layer with 10 neurons and was trained using 70% of the dataset while the rest of the data was used for testing the prediction performance. While the best results for the MW_BD_ model were attained using 7 neurons in a single hidden layer. The training process of the optimized MW_BD_ model was implemented using the 70:30 splitting ratio of the dataset for training and testing, respectively. Both models have been trained using the Bayesian regularization backpropagation (trainbr) algorithm and tan-sigmoidal (tansig) transfer function with a learning rate of 0.12.

**Figure 6:**
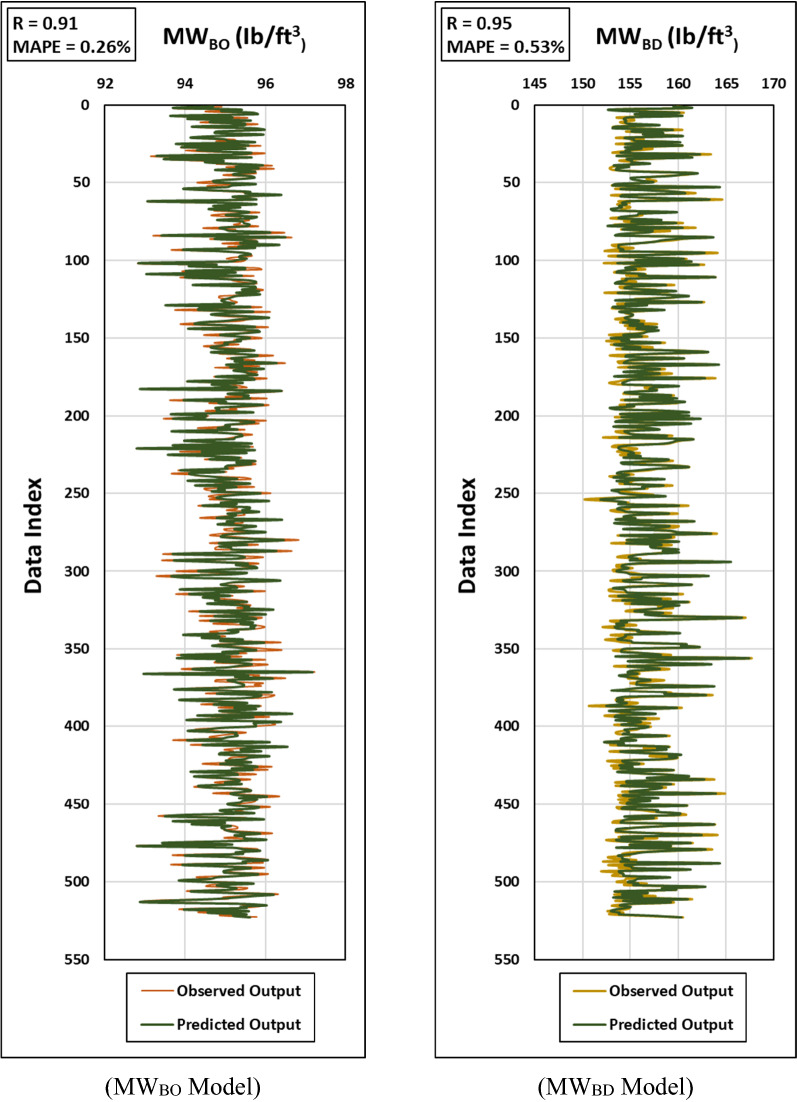
Graphical representations for the Observed vs. Predicted outputs for both MWBO (left) and MWBD (right) models for the testing process.

The selected hyper-parameters for both models are summarized in Table 3. The results showed that the models’ predictions achieved a high match between the observed and output sets indicated by R of 0.91 and 0.95 for MW_BO_ and MW_BD_ models, respectively. In terms of prediction errors, the developed models resulted in a low MAPE not exceeding 0.53% for both MW_BO_ and MW_BD_ models. The high accuracy of the developed models can be also inferred from the noticeable agreement in the graphical representation of both the actual and predicted values for the testing process as shown in Fig. 6 for MW_BO_ and MW_BD_ models.

**Figure 7:**
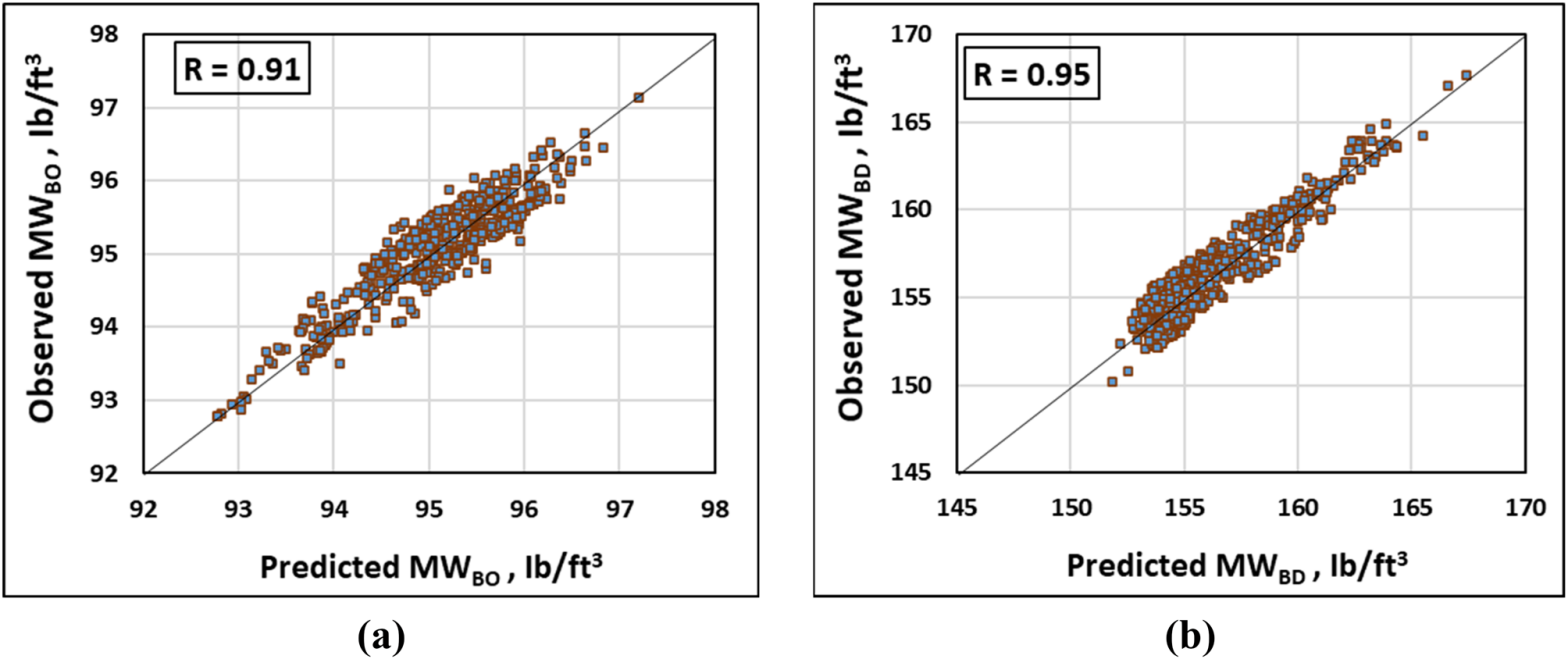
Crossplots between the actual and predicted output values for (**a**) MW_BO_ and (**b**) MW_BD_ models using the testing dataset.

Moreover, the close scatter of the observed and predicted output data for both models demonstrated a remarkable match among these values as shown in the crossplots depicted in Fig. 7. This revealed the high accuracy of the developed models’ predictions. Accordingly, the appropriate mud window for safe drilling can be defined using the MW_BO_ and MW_BD_ predictions using the developed models.

### New equations development

One of the main outcomes of this study is to develop new equations whereby the mud window for safe drilling can be determined conveniently. Therefore, new Eqs. (7) and (8) have been established using the same inputs (GR, RHOB, and DTC) to estimate both MW_BO_ and MW_BD_. The established correlations are based on the tuned weights and biases of the developed models listed in Tables 4 and 5.

**Table 4:** The optimized weights and biases of the developed ANN-based model to estimate the MWBO.

*i*	${W}_{{1}_{i,j}}$			${W}_{{2}_{i}}$	${b}_{1,i}$	${b}_{2}$
	*j* = 1	*j* = *2*	*j* = *3*			
1	− 0.146	3.033	− 0.626	0.972	− 1.907	− 0.598
2	0.136	− 0.915	− 1.555	− 2.913	− 0.630	
3	3.116	− 0.749	0.169	− 1.230	− 0.454	
4	0.587	0.957	2.175	− 0.990	0.613	
5	− 4.532	0.451	− 0.565	− 0.768	0.770	
6	− 0.593	1.210	4.230	− 0.779	2.736	
7	− 1.410	0.579	2.169	− 0.957	0.371	
8	− 0.398	2.369	− 0.515	− 1.743	− 1.829	
9	− 1.864	− 0.601	0.769	0.501	0.446	
10	1.022	− 1.752	− 0.466	1.067	− 0.439

**Table 5:** The optimized weights and biases of the developed ANN-based model to estimate the MWBD.

*i*	${W}_{{1}_{i,j}}$			${W}_{{2}_{i}}$	${b}_{1,i}$	${b}_{2}$
	*j* = 1	*j* = 2	*j* = 3			
1	− 0.445	0.393	2.084	− 0.598	0.546	1.398
2	− 0.743	− 0.797	− 0.110	1.689	− 0.369	
3	− 1.822	− 1.811	− 1.066	− 0.705	− 0.537	
4	− 0.704	1.211	− 1.903	1.188	− 1.668	
5	0.553	− 2.369	1.021	1.069	1.565	
6	− 0.266	− 1.558	− 0.526	− 1.628	1.186	
7	− 0.123	0.160	− 1.913	− 1.418	− 0.981	

7 $${MW}_{BO}=2.29 {\left({MW}_{BO}\right)}_{normalized}+95.02$$

8 $${MW}_{BD}=8.732{\left({MW}_{BD}\right)}_{normalized}+158.887$$

where, ${\left({\mathrm{MW}}_{\mathrm{BO}}\right)}_{\mathrm{normalized}}$ and ${\left({\mathrm{MW}}_{\mathrm{BD}}\right)}_{\mathrm{normalized}}$ are the normalized forms of the MW_BO_ and MW_BD_ values. The procedure required to use Eqs. (7) and (8) are described in detail in Appendix B.

### Model validation

**Figure 8:**
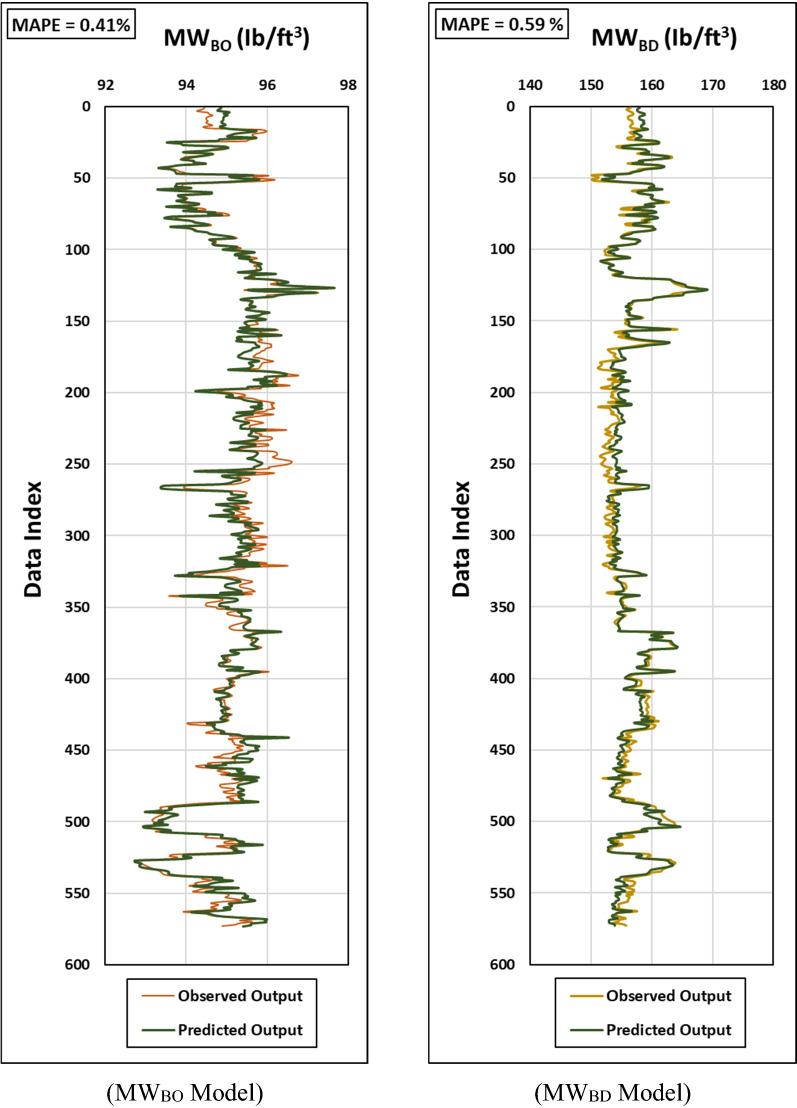
Graphical representations for the Observed vs. predicted outputs for both MW_BO_ (left) and MW_BD_ (right) models for the validation process.

In this section, the developed ANN-based equations have been validated using an unseen dataset that has not been used in the learning process. A total of 637 data points has been fed to the developed Eqs. (7) and (8) to estimate the safe mud window in terms of MW_BO_ and MW_BD_. The results have been then evaluated using R-value, MSE, RMSE and MAPE between the actual and estimated output sets. The estimated MW_BO_ and MW_BD_ showed a high agreement with the actual values as depicted in Fig. 8 with R exceeding 92% and MAPE of 0.60% at maximum. Table 6 summarizes the prediction accuracy of the developed equations in terms of MAPE, MSE and RMSE.

**Table 6:** Prediction accuracy of the developed equations to estimate MW_BO_ and MW_BD_.

	MW_BO_			MW_BD_		
	Prediction error			Prediction error		
	MAPE	MSE	RMSE	MAPE	MSE	RMSE
Training process	0.21	0.003	0.057	0.41	0.004	0.065
Testing process	0.26	0.035	0.186	0.53	0.357	0.598
Validation process	0.41	0.044	0.209	0.59	0.675	0.821

It should be highlighted that the developed correlations are more recommended for carbonate formations from which most of the data used in developing the models, were collected. This can be explained as different formation types may have different log responses and geomechanical properties. These properties control the downhole stress distributions and hence the breakout and breakdown limits. Therefore, some errors might be expected upon the application of different formation lithologies. Moreover, it is recommended to deploy the developed equations using inputs within the range and the same units listed in Table 2 to ensure reliable results.

## Conclusions

An artificial neural network (ANN) has been successfully applied to define the safe mud window limits in terms of MW_BO_ and MW_BD_. The developed models use the logging data namely GR, RHOB, and DTC as input features. The findings of this research can be summarized as follows:

- The developed ANN-based models resulted in a considerable match with the observed MW_BO_ and MW_BD_ values with accuracy exceeding 92% and a maximum mean absolute percentage error (MAPE) of 0.53%.
- New equations were established using the developed models to estimate MW_BO_ and MW_BD_ from the logging data directly without the need of running special codes.
- The developed equations were validated using unseen data from the same field. The results demonstrated the robustness of the developed equations to estimate the MW_BO_ and MW_BD_ directly from the logging data with MAPE not exceeding 0.60%.

## Supplementary Information

Supplementary Information.

## Abbreviations

ANN: Artificial neural network
MW_BO_: Minimum mud weight below which shear failure may occur
MW_BD_: Maximum mud weight above which tensile failure may occur
SMW: Safe mud window
P_f_: Formation pore pressure
P_w_: Hydrostatic mud pressure
GR: Gamma-ray logging data
RHOB: Formation bulk density log data
DTC: Compressional wave transit-time logging data
DTS: Shear wave transit-time logging data
NPHI: Neutron porosity logging data
CALI: Caliper log data
E: Elastic modulus
PR: Poisson’s ratio
UCS: Unconfined compressive strength
Ts: Tensile strength
S_rr_: Radial stress
S_θθ_: Hoop stress
S_zz_: Axial stress
β: Failure angle
trainlm: Levenberg–Marquardt
trainbr: Bayesian regularization backpropagation
trainrp: Resilient Backpropagation
trainscg: Scaled Conjugate Gradient
traincgb: Conjugate Gradient with Powell/Beale Restarts
traincgf: Fletcher-Powell Conjugate Gradient
traincgp: Polak-Ribiére Conjugate Gradient
trainoss: One Step Secant
traingdx: Variable Learning Rate Backpropagation
Tansig: Hyperbolic tangent sigmoid transfer function
Logsig: Log-sigmoid transfer function
Elliotsig: Elliot symmetric sigmoid transfer function
Radbas: Radial basis transfer function
Hardlim: Symmetric hard-limit transfer function
Satlin: Symmetric saturating linear transfer function
