# Supplementary material for: A hybrid data-driven solution to facilitate safe mud window prediction
Source: Sci Rep. 2022 Sep 21;12:15773. doi: 10.1038/s41598-022-20195-7 (PMC9492774; doi:10.1038/s41598-022-20195-7)
Supplement: Supplementary file 1 — Supplementary Information. [file 41598_2022_20195_MOESM1_ESM.doc]

# Appendix A

- **Pearson Correlation Coefficient (R-value)**

The formula used to calculate the Pearson correlation coefficient (R-value) between two variables (x and y) using a "k" number of the data points is:

| $R=\frac{k\sum xy-\left( \sum x \right)\left( \sum y \right)}{\sqrt{k\left( \sum x^{2} \right)-\left( \sum y \right)^{2}}\sqrt{k\left( \sum b^{2} \right)-\left( \sum b \right)^{2}}}$ |
| --- |

- **Mean Absolute Percentage Error (MAPE)**

| $MAPE (\%)=\frac{\sum\left\vert\frac{X_{measured}-X_{predicted}}{X_{measured}} \right\vert\times100}{n}$ |  |
| --- | --- |

where; $X_{measured}$ and $X_{predicted}$ are the actual and predicted values of the parameter, respectively, and *n* is the number of the data points.

- **Mean Squared Error (MSE)**

| $MSE=\frac{1}{n}\sum\left( X_{measured}-X_{predicted} \right)^{2}$ |  |
| --- | --- |

- **Root Mean Squared Error (RMSE)**

| $RMSE=\sqrt{\frac{1}{n}\sum\left( X_{measured}-X_{predicted} \right)^{2}}$ |  |
| --- | --- |

# Appendix B

# *Step-by-step procedure to use the new Equations (7) and (8):*

1- Transform the input data to be in the form of log(GR), RHOB and sqrt(DTC).

2- Normalize the transformed input parameters by applying the linear scaling technique using the following equations:

| $\left( log(GR) \right)_{n}=1.49 log \left( GR \right)-1.916$ | (B-1) |
| --- | --- |
| $\left( \mathrm{RHOB} \right)_{n}=3.026 RHOB-8.214$ | (B-2) |
| $\left( sqrt(DTC) \right)_{n}=1.397 sqrt \left( DTC \right)-10.349$ | (B-3) |

** where, n stands for the normalized form.*

3- Calculate the normalized forms of the MW_BO_ and MW_BD_ to substitute (MW_BO_)_normalized_ and (MW_BD_)_normalized_ in Equations (7) and (8) respectively, as follows:

- **(MW_BO_)_normalized_ Equation**

| $\left( {MW}_{BO} \right)_{normalized}=\sum_{i=1}^{k} w_{2,i}\left( \frac{1}{1+e^{-\left( \left( {w_{1}}_{i,1}\times\left( \log(GR) \right)_{n} \right)+\left( {w_{1}}_{i,2}\times{RHOB}_{n} \right)+\left( {w_{1}}_{i,3}\times\left( sqrt(DTC) \right)_{n} \right)+b_{1,i} \right)}} \right)+b_{2}$ | (B-4) |
| --- | --- |

- **(MW_BD_)_normalized_ Equation**

| $\left( {MW}_{BD} \right)_{normalized}=\sum_{i=1}^{k} w_{2,i}\left( \frac{1}{1+e^{-\left( \left( {w_{1}}_{i,1}\times\left( \log(GR) \right)_{n} \right)+\left( {w_{1}}_{i,2}\times{RHOB}_{n} \right)+\left( {w_{1}}_{i,3}\times\left( sqrt(DTC) \right)_{n} \right)+b_{1,i} \right)}} \right)+b_{2}$ | (B-5) |
| --- | --- |

where, k is the total number of neurons in the hidden layer; w_2_ is the vector of the optimized weights between the hidden layer and the output layer; w_1_ is the matrix of the optimized weights between the hidden layer and the input layer; b_1_ is the vector of the optimized biases between the hidden layer and the input layer; and b_2_ is the optimized bias between the hidden layer, the output layer. The optimized weights and biases extracted from the developed ANN-based MW_BO_ and MW_BD_ models are listed in Tables 4 and 5, respectively. This is to substitute the weights and biases in Equations (B-4) and (B-5). The input parameters should be measured in the following units: GR in API unit, RHOB in g/cm^3^, DTC in µs/ft.

4- Calculate the MW_BO_ and MW_BD_ (in Ib/ft^3^) using Equations (7) and (8) respectively.
